# The Impact of Transnational “Big Food” Companies on the South: A View from Brazil

**DOI:** 10.1371/journal.pmed.1001252

**Published:** 2012-07-03

**Authors:** Carlos A. Monteiro, Geoffrey Cannon

**Affiliations:** 1 Center for Epidemiological Studies in Health and Nutrition, School of Public Health, University of São Paulo, Brazil; 2 Editor, World Nutrition, World Public Health Nutrition Association, Rio de Janeiro, Brazil

## Abstract

In an article that forms part of the *PLoS Medicine* series on Big Food, Carlos Monteiro and Geoffrey Cannon provide a perspective from Brazil on the rise of multinational food companies and the displacement of traditional food systems, and offer suggestions for the public health response.

**Summary Points**

- Traditional long-established food systems and dietary patterns are being displaced in Brazil and in other countries in the South (Africa, Asia, and Latin America) by ultra-processed products made by transnational food corporations (“Big Food” and “Big Snack”).
- This displacement increases the incidence of obesity and of major chronic diseases and affects public health and public goods by undermining culture, meals, the family, community life, local economies, and national identity.
- The penetration of transnational companies into Brazil has been rapid, but the tradition of shared and family meals remains strong and is likely to provide protection to national and regional food systems.
- The Brazilian government, under pressure from civil society organizations, has introduced legislation to protect and improve its traditional food system; by contrast, the governments of many industrialized countries have partly ceded their prime duty to protect public health to transnational companies.
- The experience of countries in the South that still retain traditional food systems provides a rational basis for policies that protect public health.

*This article was commissioned for the* PLoS Medicine *series on Big Food that examines the activities and influence of the food and beverage industry in the health arena.*

## Introduction

Throughout human history, traditional food systems and dietary patterns have been intrinsic to social, cultural, and economic life, and to personal, community, and national identity. Although these long-established dietary patterns are rarely if ever nutritionally ideal, they are linked with low rates of obesity and chronic diseases, and can be readily improved by modifications that respect tradition and culture, and national and local resources. However, the policies and practices of transnational food and drink corporations (see Box 1), most of whose products are ultra-processed and whose headquarters are almost invariably in the US and Europe, are now steadily displacing traditional food systems around the world.

**Box 1. Big Food, Big Snack, and Ultra-Processed Products**

The term “Big Food” refers to the transnational and other large corporations that increasingly control the production and distribution of ultra-processed products throughout the world. These products are created from substances extracted from whole foods such as the cheap parts or remnants of animals, inexpensive ingredients such as “refined” starches, sugars, fats and oils, preservatives, and other additives [34],[35]. The products are formulated to be intensely palatable and to fool the body's appetite control mechanisms [36],[37]. Many of these products, while legal, are in effect fakes, made to look and taste like wholesome food. They are formulated and packaged to have a long shelf life and to eliminate the need for culinary preparation. They can be consumed anywhere, immediately or almost immediately, and often dispense with the need for tables, chairs, dishes, cutlery, and cups. They are therefore often termed “fast” or “convenience” products.

Some ultra-processed products, such as breads and sausages, have been part of dietary patterns in many countries since before industrialization. Others, such as burgers, chips, cookies, sweets, “nuggets,” energy bars, and carbonated and other sugared or sweet drinks, are more recent, at least in the quantity now manufactured. Since the 1980s, “Big Snack”—the transnational manufacturers of packaged, long shelf-life snacks designed to displace meals—have greatly increased their penetration first of high-income countries, and now of lower-income countries, including Brazil [21].

This process of displacement is not merely commercial, it is also ideological. Economic globalization, systematic privatization, and minimally regulated international capital flow have all shifted the balance between governments and corporations. Governments and international institutions now tend to cede their prime duty to protect the public interest to vast transnational corporations whose primary responsibility is to their shareholders. The prevailing political, economic, and commercial policies and practices have also given these corporations freedom to expand across borders [1]. Consequently, the leading food and drink product corporations are now colossal concerns. Their brands sell throughout the world in outlets that range from large supermarkets to filling stations, and from restaurants to kiosks. The individual annual revenue of the largest corporations is as high as the annual gross domestic product (GDP) of middle-size countries [2]–[8] and, unlike many national governments, these corporations are able to plan strategically and to divert or invest billions in new technologies and markets.

Big Food corporations now claim that they work in the public as well as the private interest, and even that they are in the business of protecting public health. For example, according to executives working for PepsiCo, food and drink transnationals penetrating “emerging markets” can at the same time support low- and middle-income countries, helping them to eliminate hunger and undernutrition and to prevent and control obesity and non-communicable diseases [9]. In our view, this claim is part of a damage limitation exercise, which is further eroding the duty of governments to protect public health and public goods [10].

The ongoing globalization, privatization, and deregulation of food systems and supplies may have relatively little impact on public health in high-income countries whose dietary patterns are already fully industrialized. But the displacement of traditional food systems in Africa, Asia, and Latin America (“the South”) by the fatty, sugary, or salty “long-life” ultra-processed products marketed by transnational food and drink corporations, which has been increasing rapidly since the 1980s, has the potential to undermine public health by increasing the incidence of chronic diseases and obesity.

We believe that the experience and situation of those countries in the South still retaining their traditional food systems provide a rational and reliable basis for the development of international public health policies related to food. In this essay, we describe the incursion of Big Food and Big Snack into the South, based upon our experiences in Brazil, a very large country that still retains, at least in part, its traditional food systems. We outline the nature of traditional Brazilian food systems and dietary patterns and the impact of the rapid penetration of the transnational food corporations into Brazil. We identify some implications of the “partnerships” between food corporations and public bodies. Finally, we propose ways to protect public health and public goods in Brazil and in other countries and regions that still retain traditional food systems and supplies.

## Traditional Brazilian Dietary Patterns

Analyses of household food expenditure surveys conducted in Brazil over the past 40 years [11]–[13] show that, in common with other Latin American countries, Brazil retains many long-established food systems and dietary patterns. These dietary patterns show the influences of native (“Indian”) populations, the country's Portuguese colonizers, and African slaves and their descendants. Minimally processed food staples include rice, a variety of beans, and the root cassava (manioc). These staples form the basis of everyday main meals, and are made delicious and attractive by various methods of preparation and cooking, and by the addition of oils, seeds, leaves, herbs, and spices, some of which are rich in nutrients. (Wheat is not native to Brazil; processed wheat products such as breads, cakes, and biscuits followed relatively recent immigration of people from the Mediterranean region to the southern states of Brazil, and nationally were uncommon until well into the second half of the last century.) The amount of meat, fish, and other animal products in long-established Brazilian diets depends on availability, price, and income. In the past, these foods were usually eaten only in small amounts on a daily basis, and in large quantities only as part of feasts or other special occasions.

All Brazilian cities have restaurants, bars, and popular canteens, where a good variety of locally sourced traditional Brazilian food is offered, often buffet-style and affordably priced on a “per kilo” basis. More importantly, meals prepared and eaten by the family at home—including the midday meal—and therefore the habit of eating together, remain an integral part of the Brazilian way of life. Notwithstanding intense pressures, which include ubiquitous television and internet propaganda designed to turn eating and drinking into constant individual snacking [10], food and drink consumption is not yet dislocated and isolated from family and social life in Brazil. This is probably the most important factor protecting national and regional traditional food systems.

It would be wrong, however, to romanticize Brazilian traditional food systems and dietary patterns—they are far from ideal. The influence of the seafaring Portuguese colonizers and the need to preserve animal foods by salting before the widespread availability of refrigeration mean that the typical Brazilian diet is high in salt, which has resulted in high rates of hypertension and stroke in the country [14]. The traditional diet is also sugary, the result of Brazil being for centuries the world's largest producer of sugar, and of table sugar being the cheapest source of calories in the country. Further, while a variety of indigenous or established tropical fruits is consumed, commonly at breakfast or as desserts, consumption of green vegetables remains low, particularly among the lowest-income families. More positively, undernutrition is generally uncommon or rare at all stages of life and, as with many Asian, African, and other Latin American countries, rates of obesity in Brazil were low until the late 1970s [15].

## Big Food and Big Snack Move In

During our work on this essay, we went for lunch to a workers' restaurant near the University of São Paulo, where a traditional freshly cooked meal of rice, beans, and a choice of meat, together with mixed salad, cost the equivalent of $US 6. We noticed that the bottled water offered was “made” by a once Brazilian company now owned by Coca-Cola, and that the artisanal water-based ice lollies containing fruit juice, which are still sold by pedlars on Brazilian beaches and supplied by traders to simple restaurants, had been replaced by fatty, sugary brands of Nestlé ice cream. These same ice creams, together with other Nestlé “popularly positioned products,” which are “targeted at and bought by low income consumers” [16], are now being sold door-to-door in the outskirts of several Brazilian cities, on trains and subway stations, in retail chains that sell electronic and house appliances, and also on “floating supermarkets” that take Nestlé products to remote Amazonian villages [10].

Our experience in the workers' restaurant illustrates how transnational food corporations are changing dietary patterns in Brazil. For these corporations, the move into emerging markets is necessary because the market for their ultra-processed “convenience,” meals, dishes, snacks, and drinks is already saturated in high-income countries [17]. Research we are undertaking suggests that in any country a saturation point is reached when ultra-processed products supply around 60% of total calories, as has been the case in the UK [18] and in Canada and the US for the last two decades (unpublished data being prepared for publication). Indeed, in the US, consumption of sugared cola and other soft drinks is now declining from a very high peak [19], and transnational corporations have moved into “designer water” and soft drinks for which health claims can be made because the drinks have been reformulated to contain more substances chemically identical to micronutrients or the dietary fibre found in whole foods.

The saturation of developed market economies with ultra-processed products may also explain why transnationals appear to be aiming for double-digit growth—sales that increase by an annual 10% or more—in the South. For instance, the growth of the Nestlé line of “popularly positioned products” is up to 25% a year, and the market for these products in Asia, Africa, and Latin America is now estimated to be over CHF 80 billion, or a little over $US 87 billion [20]. In Brazil, the consumption of ultra-processed products has already risen from less than 20% of calories in the 1980s to 28% [21], but this figure is still well short of 60%, the possible saturation level. Similarly, the current prevalence of obesity in Brazil (14% among adults in 2009) [14], has some way to go before it reaches the levels seen in countries like the UK and the US (currently 24% and 34%, respectively) [22].

More generally, the opportunities for transnational food and drink corporations to increase the penetration of ultra-processed products in very highly populated countries such as India and China, where until recently most of the populations were rural and on very low incomes, are immense. In such countries, the strategies these corporations are employing to introduce their products undermine and displace long-established traditional food systems. The impact is not only on nutrition and risk of disease. Snacking replaces meals. Commensal family and community life is undermined. Local food producers, distributors, retailers, and caterers are driven out of business. Social networks collapse. Regional and national culture and identity are eroded [23]–[26].

Epidemic obesity and serious chronic diseases can be seen as an integral part of economic development. Thus, Kenneth Rogoff, a former chief economist at the International Monetary Fund, recently wrote that: “Highly processed corn-based food products, with lots of chemical additives, are well known to be a major driver of weight gain, but, from a conventional growth-accounting perspective, they are great stuff' [27]. Given the disability caused by obesity, and the attendant diabetes, heart disease, cancer, and other diseases that need lengthy and expensive treatment, we suggest that the concept of “development” needs revision.

## Public–Private Food “Partnerships”

It is now commonly agreed in UN, government, public health, and scientific circles that the prevention and control of noncommunicable chronic diseases can be achieved only by “public–private partnerships,” meaning collaborations with “the private sector.” However, this term does not refer to industry as a whole. It is code for the food, drink, and associated industries. Indeed, in practice, the term only refers to the transnational and other large suppliers, manufacturers, and distributors of ultra-processed products and their raw materials, together with allied and associated corporations and institutions. Excluded are national industries (with a few exceptions), the retail trade (unless burger chains are counted), low-input and “organic” producers and growers, the horticulture industries (again, with a few exceptions), food and farming cooperatives, and family and smaller manufacturers, all of which have minimal or no representation in the “partnership”.

It is Big Food and Big Snack, above all, that have sought and achieved “partnerships” with the public sector. Indeed, with support from the World Economic Forum and other organisations that represent the interests of transnational industry, food corporations have already engaged in the committees convened by the UN that are tasked to set and develop the global agenda specified in the Political Declaration of the recent UN High-Level Meeting on prevention and control of noncommunicable diseases [28],[29].

Within countries where food systems are already more or less saturated with ultra-processed products, these committees are mainly focused on proposals to adjust the formulation of ultra-processed products so that they contain, for example, less salt and trans-fats or more synthetic micronutrients. But such reformulation often allows manufacturers to advertise their products as “healthy,” which is likely to result in sustained or even increased purchase and consumption of such products, additional displacement of fresh and minimally processed foods, and therefore further increases in obesity, diabetes, and other chronic diseases.

Moreover, the reformulation strategy of transnational corporations may have the effect of heading off legislation designed, for example, to sharply limit or prohibit the advertising and marketing of ultra-processed products to children. The reformulation strategy is also likely to contribute to the displacement of traditional food systems in Brazil and other countries of the South, and to the replacement of shared meals with ready-to-heat or ready-to-eat dishes and snacks labelled as healthy, often with the blessing of governments and support from nutritionists and public health professionals.

Partnership with industries whose interests do not conflict with those of public health is essential. But we see “partnerships” with Big Food and Big Snack as analogous with tobacco control or alcohol restriction policy-making in which Big Tobacco or Big Alcohol manufacturers are partners. The growth of production and therefore consumption of ultra-processed food and drink products in Brazil and elsewhere in the South is already undermining and even destroying public health and public institutions in countries that cannot afford the costs of treatment of obesity and other epidemic diseases of which unhealthy food products, consumed in typical quantities, are a major cause.

## Protecting and Improving Traditional Food Systems

The long-established Brazilian diet, in common with the diets of other Southern countries, can readily be improved from the nutritional and public health points of view. Indeed, it has already improved. Because food supplies have become secure in almost all parts of the country, and because most Brazilian populations and communities have risen up out of severe impoverishment, the consumption of meat, milk, and other animal products in Brazil has increased, undernutrition has become uncommon, and childhood stunting and wasting have ceased to be major problems [30],[31]. Remaining undernutrition can be alleviated by special programs involving redistribution of cash or goods, as carried out by recent Brazilian governments [32].

Remaining faults in the Brazilian traditional dietary patterns can be addressed by a combination of fiscal and other legislation. This legislation should support cooperatives and family farmers, protect and stabilize the prices of healthy staple foods and ingredients, and make green vegetables and other fresh and minimally processed foods more attractive to produce and more affordable and available. It should also reduce the volume of salt and sugar entering food supplies, either as raw materials or as contained in ultra-processed products [21]. Programs of information and education at the national and state levels should be used to reinforce this legislation. More generally, we also advocate worldwide prohibition of the hydrogenation process that generates industrial saturated fats and trans-fats.

The use of law to protect and improve food systems and supplies, and thus public health, may be difficult in parts of the world where governments have already surrendered the responsibility of governance to transnational and other corporations. However, in Brazil protection of public health still remains a prime duty of government that has not eroded as it has in other countries. Thus, by law, all Brazilian children are entitled to one daily meal at school, at least 70% of the food supplied to schools must be fresh or minimally processed, and a minimum of 30% of this food must be sourced from local family farmers [14]. Brazil also has more than 200 human milk banks, the largest such network in the world [31].

Two important principles used in legislation in Brazil and in some other better-resourced countries in the South add to the current conventional discourse about food, nutrition, and public health. First, access to adequate healthy food in Brazil is regarded as a basic human right that needs protection by the use of law in the public interest. The right of access to adequate healthy food has been part of the Brazilian Constitution since 2010, together with the right of access to health care, education, work, and social security [33].

Second, many improvements in Brazilian food supplies—for example, those that benefit schoolchildren, the legal support provided to working mothers to breastfeed their babies, and the prohibition of any type of advertisement of commercial human milk substitutes [31]—depend on sustained pressure on legislators exerted by energetic and often militant civil society organizations. Notably, in Brazil, partnership between government and civil society at all levels is a central feature of the 1988 Constitution, which was designed to ensure that Brazil would be a participatory democracy after its emergence from government by military regimes.

## Conclusion

This essay is based on our experience in Brazil, a large country that, together with many other countries in the South, still retains its long-established food systems and thus dietary patterns. By contrast, the traditional food systems of fully industrialized high-income countries like the US and the UK were largely displaced generations ago.

As a result, the views of many commentators and policy-makers in the South are in sharp contrast with their counterparts in the North. In countries like the US, the general tendency is to deal with food, nutrition, and public health in isolation as matters largely of information, education, and “individual lifestyle adjustments” designed to reduce the risk of various disabilities and diseases. But in Brazil and other countries in the South, food is seen by most independent scholars and policy-makers as part of a much broader discourse that involves general well-being, the family, friendship, commensality, culture, sustainable livelihoods, environmental preservation, national identity and sovereignty, as well as personal and public health.

In the North, the prevailing political and economic ideology, which has developed rapidly since the 1980s, has involved abandonment by governments of policies and actions designed to protect public health and public goods to private corporations. During this process of privatization and globalization, leading corporations have become transnationals.

Governments and other powerful and influential institutions, and also research and public health institutions, now need to examine and document the impact on the South of Big Food and Big Snack and their associated and supportive international financial and economic organizations on public health, and also on regional and national nature and culture, independence, and identity. This work must not inhibit the implementation of policies and actions that are needed now; importantly, once enacted, the impact of these essential policies and actions needs to be carefully monitored.

Brazil is one of the leading countries in the South. It has substantial remaining natural resources, and its governments at federal, state, and municipal levels remain able to govern. We propose, therefore, that the Brazilian experience provides a basis for the design of rational, comprehensive, and effective public health policies and actions designed to protect and promote nutrition in all its senses.
